# Nutrition Intervention in Gynecological Diseases

**DOI:** 10.3390/nu17132238

**Published:** 2025-07-07

**Authors:** Yuanyuan Li

**Affiliations:** Department of Nutrition and Food Science, College Park, Maryland, MD 20742, USA; roseli15@umd.edu

## 1. Introduction

Gynecological diseases are a group of diseases that affect the reproductive systems of women. These diseases include benign and malignant tumors, pregnancy-related conditions, and inflammatory and endocrine diseases that can occur in the female reproductive tract, including the cervix, ovaries, uterus, vagina, and vulva. Common gynecological diseases include endometriosis, uterine fibroids, polycystic ovary syndrome, infections, and gynecologic cancers. There are different risk factors and etiological mechanisms that are involved in the development of gynecological diseases, such as pathogenic infection, hormone changes, and immune regulation. These diseases can lead to a range of symptoms, including chronic pain, reproductive dysfunction, infertility, and cancer-related complications, all of which can profoundly impact women’s quality of life.

Globally, the health burden associated with gynecological diseases has risen significantly over the past three decades [1]. This upward trend underscores the urgent need for effective intervention strategies aimed at preventing and managing common gynecological disorders. Numerous studies have shown that nutritional factors and dietary patterns play a significant role in influencing the development, progression, and management of gynecological conditions. Nutrient imbalances, chronic inflammation triggered by poor dietary choices, and disruptions in metabolic and hormonal regulation are all recognized as contributing factors. As such, lifestyle interventions that incorporate dietary modifications and nutritional support offer promising avenues for reducing the risk and improving the clinical outcomes of gynecological diseases, leading to enhanced reproductive health, reduced healthcare costs, and improved long-term health outcomes for women worldwide.

This Special Issue comprises nine contributions and offers an overview of the advances in natural dietary patterns and interventions in gynecological disorders, including gynecological tumors and pregnancy-related conditions, inflammation and endocrine disorders. These contributions collectively underscore the translational potential of nutritional factors as both biomarkers and intervention approaches used in gynecological care.

## 2. Thematic Overview of Published Papers

Gynecologic cancers refer to any cancers that originate from a woman’s reproductive tract. Endometrial, ovarian, and cervical cancers are the top three common gynecologic cancers, collectively accounting for approximately 11% of all cancers in women [2]. Two contributions in this Special Issue discussed nutrition-oriented biomarkers and mechanisms in gynecologic cancers. Studies have shown that a healthy lifestyle, such as those that include consuming beneficial diets or nutrient supplements, is associated with a reduced risk of various cancers, including gynecologic cancers [3]. Michalczyk et al. reported the correlation between the serum levels of zinc, copper, manganese, and iron and a spectrum of endometrial pathologies, from benign conditions to malignancy. Their study reveals that the levels of specific micronutrients are altered in different endometrial pathologies, suggesting their potential roles as biomarkers in risk stratification and disease progression. Zhu et al. presented a large cross-sectional analysis using the NHANES database (2007–2016), connecting dietary intake patterns with gynecologic cancer incidence. Their findings suggest that specific nutrient combinations may influence certain gynecologic cancer risks; for example, higher intakes of phosphorus and vitamin B_12_ were associated with lower odds of cervical and endometrial cancer, while increased caffeine intake was correlated with a higher risk of cervical cancer and elevated copper intake was linked to a greater risk of endometrial cancer. These studies highlight the importance of dietary guidance in the preventive care of common gynecologic cancers.

Polycystic ovary syndrome (PCOS) is a common reproductive disorder in women of reproductive age, characterized by an irregular menstrual cycle, excess androgen levels, and ovarian cysts [4]. Women with PCOS have an increased risk of developing infertility, insulin resistance, and metabolic disorders. A lifestyle intervention emphasizing a nutrient-rich, low-glycemic diet combined with regular physical activity is the most effective approach for managing PCOS. Magagnini et al. examined the impact of a very low-calorie ketogenic diet in women with obesity and PCOS. Their findings support the evidence that ketogenic dietary approaches can improve both metabolic and reproductive health in obese women with PCOS. Uterine fibroids are a common benign neoplasm of the uterus that can cause menorrhagia, pelvic pain, infertility, and pregnancy complications [5]. Krzyżanowski et al. reviewed the current evidence linking dietary components to fibroid pathogenesis. They highlight that certain nutrients and dietary behaviors such as an increased consumption of fruits, vegetables, and vitamin D supplementation can act as protective factors influencing the incidence and growth of fibroids. In addition to PCOS and uterine fibroids, two papers in this Special Issue studied how diet and nutrition intervention can be used to manage inflammatory and endocrine-based gynecological disorders such as endometriosis. Endometriosis is a chronic inflammatory condition characterized by the growth of endometrial-like tissue outside the uterus, causing persistent pelvic discomfort and infertility in women [6]. Markowska et al. examined how specific nutrients (e.g., polyphenols, vitamins, calcium, zinc, and nickel) affect endometriosis risk and severity, advocating for evidence-based dietary interventions as adjunctive therapy. Complementing the above, Piecuch et al. provided an in-depth review of the nutritional management of endometriosis, highlighting key nutrients such as polyphenols, vitamins C, D, and E, polyunsaturated fatty acids, and iron. They also examined various dietary patterns, including Mediterranean, anti-inflammatory, vegetarian, low-nickel, and low-FODMAP diets and detailed how diet-induced inflammation and oxidative stress influence symptom severity and disease progression. Dysmenorrhea is characterized by the onset of painful menstruation occurring before or during the menstrual period, predominantly affecting adolescents and young women. Chen et al. evaluated vitamin D supplementation in relieving the symptoms of primary dysmenorrhea. The authors concluded that weekly supplementation of vitamin D, particularly doses over 50,000 IU, significantly reduced pain severity in women with primary dysmenorrhea. Although the primary treatments for dysmenorrhea are pain medications and oral contraceptives, this study suggests that vitamin D supplementation represents a promising alternative or adjunct to conventional treatments for managing this chronic gynecological condition.

Gestational diabetes mellitus (GDM) is a form of diabetes that develops during pregnancy and can increase the risk of adverse maternal and neonatal outcomes. Dietary management, particularly in terms of maintaining a healthy, balanced, and low-glycemic-index diet, is the primary nutrition guideline for GDM treatment [7]. Bankole et al. explored the links among maternal diet, the gut microbiome, the metabolome, and epigenetic mechanisms in GDM. They highlight how maternal diet-induced microbiome shifts may alter maternal/fetal metabolite profiles, leading to neonatal epigenetic reprogramming, impacting maternal–fetal outcomes, and offering targets for maternal nutrition intervention strategies in GDM patients.

Nutritional management is essential for postmenopausal women as it helps mitigate risks of osteoporosis, cardiovascular disease, and metabolic disorders while supporting overall health and well-being during this transitional stage. Lastly, Feduniw et al. reviewed the effects of vitamin E supplementation in postmenopausal women. The analysis suggests that vitamin E improves lipid profiles, alleviates menopausal symptoms, and supports vascular modulation, indicating its potential as an adjunct to hormone therapy in postmenopausal women.

## 3. Conclusions

This Special Issue compiles evidence that dietary and nutritional interventions play a critical role in preventing and managing female reproductive disorders, ranging from benign tumors to endocrine and inflammatory conditions. The nine papers collected in this Special Issue highlight that the targeted nutrient-rich strategies and dietary modifications can serve as effective adjuncts to conventional treatments in gynecological diseases, offering meaningful improvements to women’s health and quality of life. In conclusion, these findings highlight the importance of integrating nutrition-based therapies into gynecological care and call for further high-quality interventional studies in this burgeoning field.
